# Biomechanics of the human thumb and the evolution of dexterity

**DOI:** 10.1016/j.cub.2020.12.041

**Published:** 2021-03-22

**Authors:** Fotios Alexandros Karakostis, Daniel Haeufle, Ioanna Anastopoulou, Konstantinos Moraitis, Gerhard Hotz, Vangelis Tourloukis, Katerina Harvati

**Affiliations:** 1 Paleoanthropology, Senckenberg Centre for Human Evolution and Palaeoenvironment, Eberhard Karls University of Tübingen, Rümelinstrasse 23, 72070 Tübingen, Germany; 2 Hertie Institute for Clinical Brain Research and Werner Reichardt Centre for Integrative Neuroscience, Eberhard Karls University of Tübingen, Hoppe-Seyler-Strasse 3, 72076 Tübingen, Germany; 3 Institute for Modelling and Simulation of Biomechanical Systems, University of Stuttgart, Nobelstrasse 15, 70569 Stuttgart, Germany; 4 Department of Forensic Medicine and Toxicology, School of Medicine, National and Kapodistrian University of Athens, Mikras Asias Street 75, 11527 Athens, Greece; 5 Anthropological Collection, Natural History Museum of Basel, Basel 4051, Switzerland; 6 DFG Centre of Advanced Studies ‘Words, Bones, Genes, Tools’, Eberhard Karls University of Tübingen, Rümelinstrasse 23, D-72070 Tübingen, Germany

**Keywords:** evolution of tool use, 3D muscle modeling, muscle attachments, entheses, evolutionary biomechanics, functional morphology, evolution of the hand, *Australopithecus*, *Homo naledi*, early *Homo*

## Abstract

Systematic tool production and use is one of humanity’s defining characteristics, possibly originating as early as >3 million years ago.^1^, ^2^, ^3^ Although heightened manual dexterity is considered to be intrinsically intertwined with tool use and manufacture, and critical for human evolution, its role in the emergence of early culture remains unclear. Most previous research on this question exclusively relied on direct morphological comparisons between early hominin and modern human skeletal elements, assuming that the degree of a species’ dexterity depends on its similarity with the modern human form. Here, we develop a new approach to investigate the efficiency of thumb opposition, a fundamental component of manual dexterity, in several species of fossil hominins. Our work for the first time takes into account soft tissue as well as bone anatomy, integrating virtual modeling of *musculus opponens pollicis* and its interaction with three-dimensional bone shape form. Results indicate that a fundamental aspect of efficient thumb opposition appeared approximately 2 million years ago, possibly associated with our own genus *Homo*, and did not characterize *Australopithecus*, the earliest proposed stone tool maker. This was true also of the late *Australopithecus* species, *Australopithecus sediba*, previously found to exhibit human-like thumb proportions. In contrast, later *Homo* species, including the small-brained *Homo naledi*, show high levels of thumb opposition dexterity, highlighting the increasing importance of cultural processes and manual dexterity in later human evolution.

## Highlights

• Increased thumb opposition efficiency was present about 2 million years ago
• This evolutionary advantage was less pronounced in *Australopithecus*
• This dexterity is shared with all recent hominins, including *Homo naledi*

## Teaser

Karakostis et al. integrate virtual muscle modeling with three-dimensional bone shape analysis to investigate biomechanical efficiency for thumb opposition in the fossil human record. They report the earliest evidence of increased manual dexterity, a vital component of human-like tool use, in thumb bones dated to about 2 million years ago.

## Results and discussion

Manual dexterity is considered critical for the production and use of tools. Until recently, the latter was thought to have emerged approximately 2.5 million years ago (mya), closely tracking the evolution of the genus *Homo*.^1^^,^^2^ The discovery of the Lomekwian early lithic industry,^3^ as well as non-*Homo* fossil hominins bearing manual anatomical similarities to modern humans^4^ or found with early artifacts^1^^,^^2^ have challenged the perceived relationship between taxonomy, cultural shifts, and manual dexterity. Previous assessments of manual dexterity in the human fossil record have mainly relied on anatomical comparisons to modern humans and provided conflicting conclusions. Among early hominins, indications for a precision-grasping capacity, a vital component of tool making, have been reported in *Australopithecus afarensis* (dated between 3.85–2.95 mya), including a proportionally long thumb and a human-like manipulation workspace.^4^, ^5^, ^6^ The metacarpals of *Australopithecus africanus* (2.6–2.0 mya) (Table 1) exhibit a trabecular bone structure proposed to reflect forces related to precise manipulation.^7^ Furthermore, the hand of the later *Australopithecus sediba*, dated to ca. 2 mya, presents a proportionally long thumb that has been interpreted as facilitating the thumb’s opposition for human-like precision grasping.^8^ However, *Australopithecus* hand bones also show features inconsistent with high precision-grasping efficiency, such as a distinctively gracile thumb,^18^, ^19^, ^20^, ^7^, ^8^ likely indicating a limited capacity of the thumb to produce force, and a relatively primitive morphology of the lateral carpal and carpo-metacarpal joints (involving the scaphoid, trapezium, trapezoid, capitate, and metacarpals 1 to 3),^18^, ^19^, ^20^, ^7^, ^8^ possibly suggesting a low range of motion for the trapezio-metacarpal (TMC) joint^4^^,^^7^^,^^8^^,^^10^ (see also a previous biomechanical study^11^). Among later hominins, hand bones variably attributed to *Paranthropus* and early *Homo* species have previously been associated with human-like tool making capacities.^5^^,^^9^, ^10^, ^11^, ^12^, ^13^, ^14^

**Table 1:** List of specimens used in the biomechanical models and their general characteristics

Species / population	Trapezium sampled	Specimen(s)	Sex	Location	Date
*Australopithecus afarensis*	X	A.L. 333-80 / A.L. 333-w39	Undetermined	Eastern Africa	ca. 3.85–2.95 mya
*Australopithecus africanus*		StW 418	Undetermined	South Africa	ca. 2.6–2.00 mya
*Australopithecus sediba*		Malapa Hominin 2	Female	South Africa	ca. 1.98 mya
Early *Homo* or *Australopithecus robustus* (Swartkrans)		SK 84	Undetermined	South Africa	ca. 2.19–1.80 mya
		SKX 5020	Undetermined	South Africa	ca. 2.19–1.80 mya
*Homo naledi*	X	Hand 1	Undetermined	South Africa	335–236 thousand years (ka)
Neanderthals	X	Shanidar 4	Male	Near East	100–75 ka
	X	Kebara 2	Male	Near East	64–56 ka
	X	La Ferrassie 1	Male	Western Europe	45–43 ka
	X	La Ferrassie 2	Female	Western Europe	45–43 ka
Early *Homo sapiens*	X	Qafzeh 9	Female	Near East	130–92 ka
	X	Ohalo 2	Male	Near East	ca 23 ka
Recent *Homo sapiens*	X	Basel-Spitalfriedhof Collection	5 Males	Central Europe (Switzerland)	19th century
*Pan troglodytes*	X	Osteological collection (Natural History Museum of Basel)	3 Females, 2 Males	Central Europe (zoological garden)	20th century

Please see Karakostis et al.,^18^ Kivell et al.,^19^ and the Wiley-Blackwell Encyclopedia of Human Evolution.^20^

Several of these studies have focused on morphological characters with extensive functional significance,^4^^,^^8^^,^^9^^,^^21^^,^^22^ providing novel insights into hominin behavior on the basis of variation in the three-dimensional (3D) form of the bone’s external aspects^18^^,^^9^^,^^10^^,^^22^ or their underlying trabecular structures (e.g., Kivell^4^ and Dunmore et al.^21^). However, most of this previous research has typically relied on comparative anatomical analyses, without directly quantifying grasping efficiency biomechanically (as, for example, in Feix et al.^6^ and Domalain et al.^11^) and has not always focused on the thumb,^14^^,^^15^ the central component of precision grasping, crucial in exerting and resisting forces during tool manipulation.^4^^,^^16^^,^^17^ Most importantly, hand remains from Swartkrans, South Africa, dated to ca. 2.0–1.8 mya, have been interpreted as supporting tool-making capabilities for *Paranthropus robustus*.^12^ However, their taxonomic attribution remains uncertain because both early *Homo* and *P. robustus* occur at this site during this period.^4^^,^^13^ These hand bones present several distinctive human-like attributes^10^^,^^13^ (but see Marzke et al.^10^ regarding the more chimpanzee-like curvature of the trapezial facet in metacarpal SK84). Most past interpretations of the manipulatory capabilities of fossil hominin hand bones have therefore depended on the assumption that their level of manual dexterity is directly related to the degree to which they resemble the modern human form. However, this premise neglects the fact that a similar level of biomechanical efficiency can be achieved by structures with distinct morphologies^23^ and does not address the critical influence of soft tissues (e.g., muscle properties) on grasping performance (as in Synek et al.^24^ and van Leeuwen et al.;^25^ also see examples from the bio-medical literature^26^, ^27^, ^28^, ^29^).

### Modeling thumb opposition efficiency (torque) in modern humans and chimpanzees

Here, we use an integrative approach for investigating manual dexterity in the fossil record based on joint torque, a fundamental indicator of biomechanical efficiency (see STAR methods). Essentially, the objective of the present study is not to reconstruct habitual physical activity patterns in early hominins, but to employ an integrative biomechanical approach for detecting key functional adaptations for increased manipulatory skills in the fossil record. Through the integration of muscle modeling in 3D and geometric morphometric shape analysis, our methodology considers the crucial effects of muscle parameters (i.e., force-producing capacities) and bone morphology at the sites where muscles attach in life.^18^^,^^30^, ^31^, ^32^ In contrast to previous research, we strive to focus on anatomical structures that are functionally equivalent across extinct hominin species by evaluating only features and actions that are present in both extant humans and species of the genus *Pan*, our closest living relatives.^33^^,^^34^ We chose chimpanzees as our comparative sample because of their phylogenetic proximity to hominins, but also because the biomechanics of their hand muscles (including joint torques) have been adequately investigated in previous anatomical and experimental cadaveric studies, allowing for valid inter-species comparisons of functionally equivalent structures.^33^, ^34^, ^35^, ^36^, ^37^ We model contraction of *m. opponens pollicis*, a muscle of vital importance for thumb opposition, whose location, pathway, and general areas of attachment are equivalent in both taxa, and among great apes in general^33^^,^^34^ (but see Method details, for considerations regarding the muscle’s insertion area). Furthermore, we focus on a specific thumb action (i.e., flexion at the TMC joint), for which *m. opponens pollicis* exhibits the same function and direction of forces in both extant humans and chimpanzees^34^ (Video S1) (for other thumb actions of this muscle see Marzke et al.^34^ and STAR methods).

**Figure 1:**
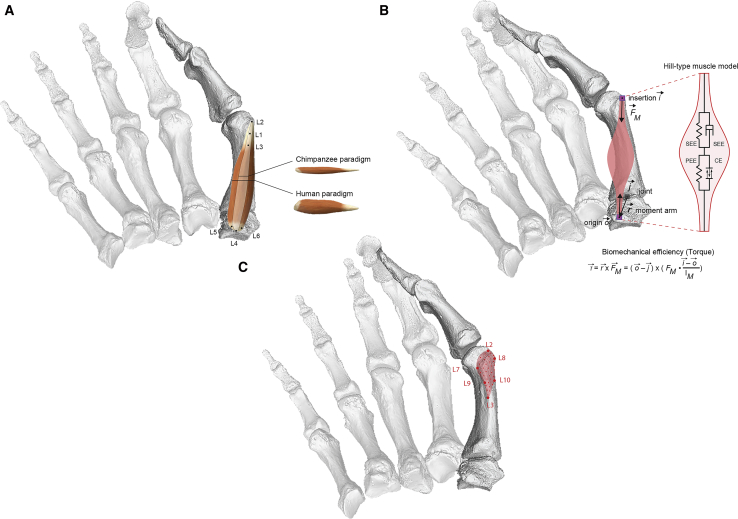
Summary of the study’s analytical steps (A) Model preparation (see STAR methods; for landmark definitions, see Table S4) and assumption of either human or chimpanzee muscle force-generating capacity (*m. opponens pollicis*). (B) Biomechanical efficiency is calculated as the torque generated by *m. opponens pollicis* at the thumb’s TMC joint (see Video S1, Table S3, and Figure S1B). The torque depends on the location of origin and insertion and, thus, on the selected enthesis’ landmark (also see Tables S4–S6). The torque further depends on the muscle force (F_M_,) which was calculated on the basis of a Hill-type muscle model^42^ (also see next section below). This muscle model has four elements, as follows: the contractile element (CE), representing the muscle fibers; the parallel elastic element (PEE), representing the connective tissue within the muscle belly; the serial elastic element (SEE); and the serial damping element (SDE) (see also Table S2 presenting muscle parameters). Both SEE and SDE together represent mainly the visco-elastic properties of the tendon. In this study, only a static position is investigated for which the muscle force F_M_ is only influenced by the physiological cross-sectional area (PCSA), and therefore only differs between the human or chimpanzee paradigm (Figure 1A). (C) 3D geometric morphometric analysis of proportional bone projection across the metacarpal muscle attachment site (see landmark descriptions in Table S4 and 3D shape analysis in Figure S1A).

**Figure 2:**
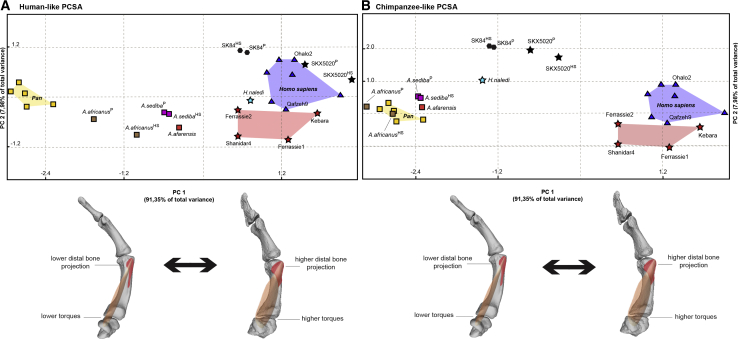
Plots of the principal component analyses based on three torque variables and relative bone projection Plots of the PCAs were based on three torque variables (see Table S3, Figure S1B, and Video S1) and relative bone projection at 3D areas of muscle attachment (see Figure S1A and Table S4), under the assumption of either a human (A) or chimpanzee (B) muscle force-generating capacity for the earlier hominins (see relevant statistics in Table S1 and a summary of the biomechanical modeling procedure in Video S1). The underlying figures represent differences related to the main axis of variation (PC1). The analysis includes modern humans (blue triangles), Neanderthals (red stars), *Homo naledi* (light blue star), *Australopithecus* (rectangles), the two Swartkrans specimens (black symbols), and chimpanzees (yellow rectangles). In specimen labels, the superscript “P” indicates that a chimpanzee trapezium was used in the model, whereas the superscript “HS” refers to the use of a modern human trapezium (see STAR Methods). The results of the repeatability analysis are presented in Figure S1C.

**Figure 3:**
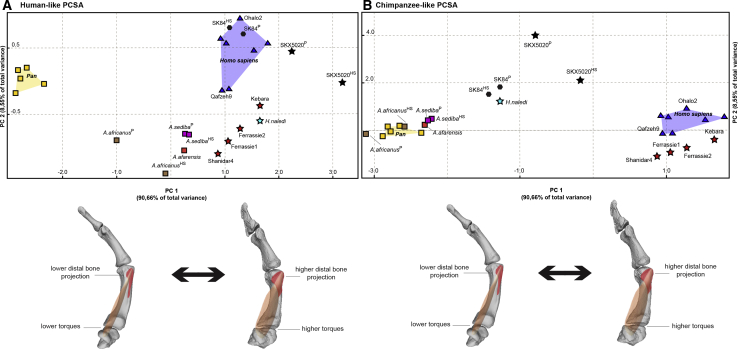
Plots of the principal component analyses based on size-adjusted torque calculations Plots of the principal component analyses based on three size-adjusted torque variables (see Table S3, Figure S1B, and Video S1) and relative bone projection at 3D areas of muscle attachment (see Figure S1A and Table S4), under the extreme assumptions that earlier fossil hominins exhibited either a mean human (A) or chimpanzee (B) muscle force-generating capacity (see relevant statistics in Table S1 and a summary of the biomechanical modeling procedure in Video S1). The underlying figures represent differences related to the main axis of variation (PC1). The analysis includes modern humans (blue triangles), Neanderthals (red stars), *Homo naledi* (light blue star), *Australopithecus* (rectangles), the two Swartkrans specimens (black symbols), and chimpanzees (yellow rectangles). In specimen labels, the superscript “P” indicates that a chimpanzee trapezium was used in the model, whereas the superscript “HS” refers to the use of a modern human trapezium (see STAR methods). The results of the repeatability analysis are presented in Figure S1C.

Video S1. The thumb posture analyzed in the biomechanical modeling procedure (contraction of *m. opponens pollicis* for flexion at the TMC joint), related to Figures 1, 2, and 3 This study’s approach focuses on the calculation of joint torque by using different muscle paradigms (force-producing capacities) as well as landmark locations on the muscle attachment bone sites. The subsequent statistical analyses combined the resulting torque calculations with the degree of three-dimensional bone projection across the entire metacarpal enthesis (Figure S1).

Even though our models rely on the function of a single muscle and joint, the associated thumb placement (Figure 1; Video S1) constitutes a fundamental step for any type of precision grasping during human tool-use,^4^^,^^5^ as well as for many types of chimpanzee food manipulation.^36^ Moreover, *m. opponens pollicis* is widely considered to have played a central role in the evolution of human dexterity^5^, ^6^, ^18^^,^^9^^,^^10^^,^^30^^,^^42^, ^38^, ^39^, ^40^ (for a discussion on the other thenar muscles, see STAR methods). The equivalent nature of the structures involved in this crucial thumb movement offers a rarely established scientific basis for approaching the evolution of hominin manual dexterity in a comparative fashion.^33^ We verified the validity of our biomechanical models by demonstrating that the resulting mean torque differences between humans and chimpanzees closely agree with those recorded during past cadaveric experiments for the same muscle, joint, and thumb movement^34^ (also see “Model precision and validation” in STAR methods). These interspecies differences are also reflected in our statistical analyses, which demonstrate a clear distinction between chimpanzees and modern humans (Figures 2 and 3).

### New insights into the evolutionary history of human thumb opposition

In our principal component analyses (PCAs), those individuals with positive scores on principal component 1 (PC1) (which explains more than 90% of total sample variance) exhibit higher joint torque values combined with proportionally more projecting insertion sites for *m. opponens pollicis* (Table S1; Figure S1A) than did those with negative scores. Variation on PC2 (representing less than 10% of total variance) depends on differences among specimens in the proportion between the degree of the muscle attachment’s bone projection and overall joint torque (see factor loadings in Table S1). We focused our interpretations on PC1, given that it explains an overwhelming proportion of sample variance (see details in STAR methods). For Neanderthals and early modern humans, we assumed muscle force production capacities for *m. opponens pollicis* similar to those of modern humans, on the basis of the genetic and cultural similarities between these two taxa. For all other fossil hominins, we ran the model assuming two different muscle force-production capacities, corresponding to (1) modern humans, and (2) chimpanzees (see muscle parameters in Table S2). Given that the actual muscle forces of these fossil hominin species are unknown, these model parameters can provide an indication of how the efficiency (torque) of each early hominin might vary when assuming distinct force-producing capacities (also see Synek et al.^24^ and van Leeuwen et al.^25^).

In the PCA plots, early modern humans and Neanderthals broadly overlap with recent modern humans, presenting positive scores on PC1, in agreement with the current consensus on their manual capacities.^4^, ^5^, ^6^ These results confirm that, if we assume that muscle force-producing capacities were not extensively different between Neanderthals and modern humans (see STAR methods), then skeletal differences would not lead to considerable torque variation between the species.^6^ When assuming a modern human-like force-generating capacity (Figure 2A), all *Australopithecus* taxa plot between modern humans and chimpanzees, whereas *H. naledi* and one of two specimens from Swartkrans (SK84) overlap with Neanderthals. Remarkably, the other Swartkrans specimen, (SKX5020), is the only early hominin in our sample plotting within the modern human range of variation under this assumption. When assuming an average chimpanzee force-producing capacity (Figure 2B), the PC1 values of all early hominins become more negative. In this scenario, all *Australopithecus* specimens, including *A. sediba*, plot either near or within our chimpanzee range. In contrast, *H. naledi* and the two Swartkrans specimens show distinctly more positive values than chimpanzees, plotting approximately halfway between chimpanzees and modern humans in the PCA.

We accounted for the potential effects of overall size on our results by running the same biomechanical models after size adjustment based on uniform scaling (see STAR methods). Results remained largely the same, but nevertheless revealed some interesting new patterns (Figure 3): when size differences are accounted for, the degree of overlap between Neanderthals and modern humans on PC1 increases; the efficiency scores of *H. naledi* and the two Swartkrans specimens also increase, and *A. sediba* shows a higher efficiency than the other *Australopithecus* or chimpanzee specimens (Figure 3). When assuming a chimpanzee-like force-producing capacity for *A. sediba* (Figure 3A), its difference to chimpanzees is visible but comparatively limited. Nonetheless, even in the scenario in which *A. sediba* had already developed the high force-generating capacities of modern humans (Figure 3B), its efficiency values for thumb opposition would still be more comparable to those of earlier *Australopithecus* species either with or without size-adjustment (Figure 3; Table S3; also see Figure S1B summarizing mean differences in torque values among groups and/or specimens), despite certain modern human-like features of its thumb and wrist.^8^ In contrast, our observations on the Swartkrans specimens appear to be consistent with their several human-like traits^10^^,^^13^ (but see some of the results reported in Marzke et al.^10^). Furthermore, our results show substantial opposition efficiency in *H. naledi*, supporting recent conclusions about this species’ manual dexterity and possible tool use^19^ (but also see subsection “Methodological limitations,” in STAR methods).

This study’s findings show that *Australopithecus*, including the late species *A. sediba*, was characterized by comparatively low joint torque values associated with *m. opponens pollicis* and flexion at the TMC joint. Essentially, even in the relatively unlikely case that its *m. opponens pollicis*’ architecture was similar to that of recent modern humans, its skeletal morphology would not permit a modern human-like level of opposition efficiency (torque). Although our results on thumb opposition do not reject possible tool production and use by these taxa or the broader ability of *Australopithecus* to perform precision grips, we show that their efficiency for this fundamental component of human-like dexterity (i.e., TMC torque) would have been consistently lower than that shown by Pleistocene *Homo*. Our results further indicate that an increase in this key aspect of manual dexterity occurred ca. 2.0–1.8 mya in some (Swartkrans), but not all (*A. sediba*), hominins from this time period. This shift potentially represented a significant evolutionary advantage, which might have been part of the crucial bio-cultural developments taking place after 2 mya. These include the emergence of the relatively large-brained *H. erectus s.l.* lineage,^43^^,^^44^ a habitual biped with increased body mass and reduced dentition, as well as the emergence of derived subsistence strategies, such as systematic animal butchery, persistent hominin carnivory, and the use of aquatic resources, which do not acquire a strong archaeological signal until after 2 mya.^43^^,^^45^^,^^46^ Stone tool use acquires a habitual dimension from this point onward, suggesting a tool-assisted widening of the dietary niche, described as a grade-level shift to an adaptive zone marked by an increasing mediation of technology.^47^^,^^48^

The two Swartkrans specimens, which show the earliest biomechanical evidence of highly efficient thumb opposition in our sample, have previously been variably attributed to early *Homo* or *Paranthropus*. They were recovered in association with the oldest evidence of hominin butchery of large vertebrates in South Africa, with one of the oldest records of hominin early access to carcasses,^45^ and with some of the earliest known bone-tool shaping and use.^2^ Our findings therefore suggest that a high level of manual control might have co-evolved with (or was exapted for) extractive foraging behaviors, which would in turn have stimulated advances in grasping capacities, in tandem with shifts in hominin technology. Our results therefore indicate yet another notable similarity between the Swartkrans hand fossils and *Homo*.^10^^,^^13^ However, it is important to note that their conclusive taxonomic identification—and elucidation of the manual capabilities of Paranthropus—can only be achieved through a secure association of these hand bones with diagnostic elements, such as craniodental remains, belonging to one or the other taxon.

All later *Homo* taxa examined here maintained—or independently developed—a high level of thumb opposition dexterity, attesting to the adaptive significance of this functional trait. Our results on *H. naledi* provide biomechanical support for previous morphological analyses of this species’ hand skeleton, which reported indications of tool-using manual capacity.^19^ Although no artifacts have been found in association with this taxon as yet, such enhanced manual abilities in this small-brained species suggest a decoupling of the traditionally assumed correlation between brain size and tool-using skills in the fossil record and therefore a potential greater importance of brain complexity in cultural behavior.^49^ Finally, the similar efficiencies observed in the derived thumbs of Neanderthals and modern humans (Figures 2 and 3) suggest that these species likely inherited this evolutionary asset from dexterous common ancestors, whose developed manual skills set the functional foundations for the accelerated biocultural evolution of recent *Homo*.

Our analysis focused on the function of a thumb muscle and joint crucial for tool production and use. Future investigation of additional key muscles of the thumb as well as the other rays (see subsection “Methodological limitations,” in STAR methods) will lead to more holistic biomechanical analyses of overall hominin hand function and shed light on whether biomechanical solutions involving other regions of the hand (e.g., the hypothenar muscles) might have complemented—or compensated for—the thumb opposition efficiencies calculated here. Moreover, even though this study focused explicitly on joint torque, the observed interspecies differences might potentially be associated with variation in fingertip force. This possibility seems to be supported by our calculations of “torque relative to thumb length” (TTL), which broadly reflect this study’s overall observations (see two rightmost columns of Table S3). In fact, this variable seems to present even higher values for *Homo naledi*, in line with the above interpretations regarding that species’ dexterity.^19^ This finding encourages future biomechanical research to incorporate additional and more complex aspects of thumb morphology, which are needed to further investigate the functional significance of the torque differences revealed here. Finally, due to the fact that there is no association between the physiological cross-section areas (PCSA) of *m. opponens pollicis* and the size of first metacarpals in extant species (i.e., modern humans and chimpanzees exhibit very similar mean first metacarpal lengths^50^^,^^51^ but extensively different mean PCSAs for *m. opponens pollicis*^34^), our biomechanical models were not able to consider whether and how the parameters of that muscle might scale with bone size in the fossil record. In the future, identifying such potential allometric associations between skeletal size and the PCSA of *m. opponens pollicis* could further refine the predictions of biomechanical modeling (also see STAR methods).

In summary, our results provide biomechanical evidence that, approximately 2 mya, certain hominins developed greatly increased thumb opposition efficiency (joint torque) relying on *m. opponens pollicis*. This crucial evolutionary advantage, which is shared with all later species of *Homo*, was found to be less pronounced in the earliest proposed stone-tool-making hominins (i.e., *Australopithecus* species, including the late *Australopithecus sediba*). The increased thumb opposition efficiency shown by all Pleistocene *Homo* species investigated here highlights the significance of this functional feature in the bio-cultural evolution of our genus.

## STAR★Methods

### Key Resources Table

**Table:** 

REAGENT or RESOURCE	SOURCE	IDENTIFIER
**Biological Samples**		

Three-dimensional models of fossilized first metacarpal and trapezium of *Australopithecus afarensis*	ARCHH (Ethiopia) and the Max Planck Society in Germany	Cat#AL333-80; Cat#AL333-w39
Three-dimensional models o fossilized first metacarpal of *Australopithecus africanus*	University of the Witwatersrand, South Africa	Cat#StW418
Three-dimensional models of fossilized first metacarpals from Swartkrans (early *Homo* or *Australopithecus robustus*)	Ditsong National Museum of Natural History, South Africa	Cat#SK84; Cat#SKX5020
Three-dimensional models of fossilized first metacarpals and trapezium of Neanderthal individuals from Israel	Tel Aviv University, Israel	Cat#Shanidar4; Cat#Kebara2
Three-dimensional models of fossilized first metacarpals and trapezia of Neanderthal individuals from France	National Museum of Natural History (Paris, France)	Cat#LaFerrassie1; Cat#LaFerrassie2
Three-dimensional models of fossilized first metacarpals and trapezia of early modern human individuals from Israel	Tel Aviv University, Israel	Cat#Qafzeh9; Cat#Ohalo2
Three-dimensional models of fossilized first metacarpal and trapezium of *Homo naledi*	Evolutionary Studies Institute (Johannesburg, Gauteng, South Africa), http://www.morphosource.org	Cat#Hand1
Recent *Homo sapiens* hand first metacarpals and trapezia	Natural History Museum of Basel, Switzerland	Cat#285; Cat#324; Cat#211; Cat#106; Cat#9
*Pan troglodytes* hand first metacarpals and trapezia	Natural History Museum of Basel, Switzerland	Cat#7943; Cat#10824; Cat#7942; Cat#8869; Cat#10913

**Deposited Data**		

Joint torque calculations and three-dimensional bone shape data	This paper	https://doi.org/10.5061/dryad.fttdz08rs

**Software and Algorithms**		

Avizo v. 9.2.0 Lite	Visualization Sciences Group	https://www.fei.com/software/avizo3d/%C2%A0#gsc.tab=0
Geomorph v. 3.3.1 (R-CRAN)	Adams and Otárola et al.^53^	https://CRAN.R-project.org/package=geomorph
MATLAB/Simulink (2019a)	MathWorks	https://www.mathworks.com/products/simulink.html
SPSS v. 24	IBM Inc.	https://www.ibm.com/analytics/spss-statistics-software
PAST v. 4.03	Hammer et al.^54^	https://palaeo-electronica.org/2001_1/past/issue1_01.htm

**Other**		

Muscle model	This paper	https://github.com/daniel-haeufle/macroscopic-muscle-model

### Resource Availability

#### Lead Contact

Further information and requests for resources and reagents should be directed to and will be fulfilled by the Lead Contact, Katerina Harvati (katerina.harvati@ifu.uni-tuebingen.de).

#### Materials Availability

This study did not generate new unique materials.

#### Data and Code Availability

Original data have been reposited to Dryad: https://doi.org/10.5061/dryad.fttdz08rs. The developed muscle model is open-source available here: https://github.com/daniel-haeufle/macroscopic-muscle-model.

### Experimental Model and Subject Details

Our biomechanical models relied on first metacarpals and trapezia from a total of 22 individuals, including extant modern humans (*Homo sapiens*, n = 5) and chimpanzees (*Pan troglodytes verus*, n = 5), as well as a large number of Plio-Pleistocene fossil hominins (Table 1). Although this sample size is relatively small, it is much larger than the one used in previous research on complex biomechanical models involving 3D bone geometry, joints, and different muscle parameters.^11^ Although small, it allows the consideration of individual variation in our model estimations. The mean torque calculations of our virtual models for these groups closely agree with those reported in former experimental analyses on hand cadavers^34^ (see below section “Model precision and validation”). Our chimpanzee sample comprised the right trapezia and first metacarpals of five non-pathological adult individuals (3 females and 2 males) curated at the Museum of Natural History in Basel, Switzerland (see Acknowledgments). Permission for their analysis was granted by the Natural History Museum of Basel, which is legally responsible for the conservation and scientific study of these skeletal remains. Our modern human sample comprised the right trapezia and first metacarpals of five adult male individuals from the uniquely documented Basel-Spitalfriedhof collection (Natural History Museum of Basel, Switzerland),^18^^,^^55^ as well as two fossil modern human adults from Israel: a female dating to approximately 100-92 thousand-years-ago (ka) (Qafzeh 9) and a male from ca. 23,000 ago (Ohalo 2).^18^ Despite the wide geo-chronological range of our modern human sample (Table 1), we did not observe considerable biomechanical differences in efficiency across modern human specimens (see torque grand means in Table S3 and Figure S1B; also see Figures 2 and 3). This is in line with previous biomedical literature on living human populations, which observed low sexual dimorphism in morphological and/or functional aspects of the TMC joint (see Schneider et al.^56^ and references therein).

Our fossil hominin samples further included *Homo neanderthalensis*, *Homo naledi*, *Australopithecus afarensis*, *Australopithecus africanus*, *Australopithecus sediba*, and two specimens from Swartkrans (South Africa) variably attributed to early *Homo* or *P. robustus* (Table 1). The Neanderthal sample involved four individuals with adequate preservation of first metacarpals and associated trapezia. For *H. naledi,* we used the thumb bones of the almost completely preserved “Hand 1” skeleton.^19^ The earlier hominin sample was composed of specimens from Hadar, Ethiopia (*A. afarensis*), Sterkfontein, South Africa (*A. africanus*), Malapa, South Africa (*A. sediba*), and Swartkrans, South Africa (SK84 and SKX5020). Among these, only *A. afarensis* preserves a trapezium (for more information on its preservation status, see the next sections). Therefore, the remaining early hominin species are represented in this study only by their metacarpal bone (for more information on addressing this issue in our models, see section below). For several fossils, (i.e., the trapezium from Hadar as well as the thumb bones of Ohalo 2, Shanidar 4, Kebara, Sterkfontein, and SK84), due to poor preservation or missing bones in the right anatomical side, the analyses were based on mirrored versions of the left bones. The inclusion of these mirrored specimens did not affect the resulting patterns per group and did not affect measuring precision (see last section of Methods). Finally, it should be mentioned that the fossil remains of certain other early fossil hominins could not be included in this study due to poor preservation of the thumb bones or of their muscle attachment sites (e.g., *Homo habilis* and *Paranthropus boisei*), not fully developed hand bone morphology (*Homo erectus* specimen KNM-WT-15000), or accessibility (*Ardipithecus ramidus* or *Australopithecus prometheus*). For the geometric morphometric analysis of entheseal 3D shape, sample information is provided below (under section “Quantifying 3D bone projection").

### Method Details

#### Grip selection and model preparation

All analyses were conducted using high-resolution 3D surface scans of the two thumb bones, which were obtained using structured-light, laser, or micro-computed tomography scanning. We have previously verified that the inter-method error in the representation of hand bone morphology is negligible^18^ (also see other studies with agreeing results^58^, ^59^, ^60^, ^61^). For each specimen, the two developed 3D meshes were exported in STL format and imported into the software package Avizo (version 9.2.0 Lite, Visualization Sciences Group), in order to be placed at the appropriate positions for the modeled thumb action (Figure 1).

The analyzed thumb posture involves flexion at the TMC joint of the thumb (Video S1). This movement, which brings the thumb toward the palm and fingers, represents a vital prerequisite for the precise manipulation of objects placed between the thumb and the index finger (e.g., fine grips) or within the palm and sustained by the fingers (e.g., three-jaw chuck grips).^5^ It is therefore considered as necessary for almost all tool-related activities in humans,^4^^,^^5^^,^^17^^,^^61^ as well as for basic food-processing actions in chimpanzees.^35^^,^^36^ Importantly, experimental research has shown that this specific thumb action (flexion at the TMC joint) is associated with a function of *m. opponens pollicis* (i.e., a direction of forces and resulting thumb movement) that is equivalent between humans and chimpanzees, our closest living relatives.^34^ It should be noted that, for other thumb movements, the function of this muscle is different between chimpanzees and humans (i.e., it acts as an abductor in humans but an adductor in chimpanzees).^34^ Furthermore, unlike several (but not all) other hand muscles, *m. opponens pollicis* exhibits corresponding muscle pathway and general location of the attachment areas across extant great apes^33^^,^^34^ (but see section “Quantifying 3D bone projection") for considerations regarding its insertion area). On this basis, the likelihood that these structures were also functionally equivalent in extinct hominins is very high, offering the necessary scientific framework for meaningful comparisons and functional interpretations across species. In fact, the entheses of *m. opponens pollicis* have been frequently analyzed in past anthropological research,^39^^,^^40^^,^^62^^,^^63^ likely due to their high distinctiveness and morphological variability across and within hominin species. In contrast, given that *m. flexor pollicis brevis* and *m. abductor pollicis brevis* tend to insert into the same broader tubercle of the proximal phalangeal base,^41^ an accurate distinction of each muscle’s attachment area on the fossil remains of extinct species would be challenging. Importantly, modeling these muscles’ TMC torque in our samples would require an adequate preservation of three consequent thumb bones in each fossil hominin (trapezium, metacarpal, and proximal phalanx), which would lead either to the exclusion of important specimens in our study (e.g., the two Swartkrans metacarpals) or the introduction of considerable bias. Finally, the remaining thenar muscle, *m. adductor pollicis*, does not contribute to TMC flexion in chimpanzees.^34^

Initially, the 3D meshes of the first metacarpal and trapezium were virtually placed in anatomical position, with the basal articular surface of the metacarpal facing the distal articular surface of the trapezium (Figure 1). Then, we centered (brought together) the two opposing bones at the central points of their articular surfaces (i.e., the entire articular facet of each bone, including its outline edges), defining central points as the geometric centers of these surface areas (computed using the measurement tools of the Avizo software). Third, we rotated the metacarpal until the borders of the adjoining articular surfaces were interlocked at a relaxed thumb position. This step relied on visual assessment of the two articulating surfaces’ outline shape, which is also influenced by the curvature of the joint (e.g., see Galleta et al.^9^ and Marzke et al.^10^). Subsequently, the distance between the two central articular points was increased to 1.5 mm for all specimens (Figure 1A). This value, which represents the modern human average thickness of cartilage in the TMC joint (both bones taken together),^64^ was used as a proxy of intra-articular space between the bones. It should be clarified that our models assumed uniform cartilage thickness at joints, despite the fact that previous research has shown that this varies across the articular surface.^65^ A very similar value (1.56 mm) was also found in the cadaveric hand specimen of a chimpanzee curated in the Natural History Museum of Basel (Table 1). To obtain that measurement, this individual was scanned using a micro-computed tomography scanner in the University of Basel (see Acknowledgments) and intra-articular joint space was then computed in the software Avizo. It is worth noting that the resulting distance between the two surfaces was influenced by their depth and, therefore, the degree of joint curvature (e.g., see Galleta et al.^9^ and Marzke et al.^10^). Finally, the metacarpal was flexed (in the palmar direction of the bones) onto the trapezium’s articular surface at 11 degrees (Figure 1A), resulting in a more medial position for the first metacarpal. Based on our direct measurements in the developed virtual models of the present study (especially those with well-preserved first and second hand rays; e.g., see Figure 1), this level of slight flexion at the TMC joint, which corresponds to approximately one third of the average maximum angular excursion for TMC flexion in great apes (32.8 degrees) and humans (37.6 degrees) ^52^^,^^63^, brings the thumb to a position of potential interaction either with the index finger (e.g., for fine grasping of small objects) and/or the remaining fingers (e.g., for precise manipulation of relatively sizeable objects held at the palm) (see Figure 1B; Video S1). We additionally confirmed these characteristics of our selected thumb posture (i.e., bone positioning; Figure 1 and Video S1) through direct observations and angle calculations on chimpanzee and modern human hand skeletons with preserved joint soft tissue, which were provided by the Natural History Museum of Basel and the Medical School of the National and Kapodistrian University of Athens, respectively (see Acknowledgments). Even though the required degree of thumb flexion may depend on the size of the object manipulated,^66^ previous experimental work has demonstrated that the moment arm of *m. opponens pollicis* for flexion at the TMC joint exhibits limited variation across the joint’s angular excursion (i.e., over a range of 20 degrees, this muscle’s average moment arm ranges between 12.3 and 12.9 mm; see Smutz et al.^29^). This very low variability in muscle moment arm indicates that greater or lesser flexion would not considerably affect our torque calculations and resulting patterns (Figures 2 and 3; Table S3).

Most early hominins do not preserve trapezia. To estimate the potential maximum error of this unknown parameter, we followed previous research^6^ and took advantage of the pronounced morphological difference of the trapezium between modern humans and chimpanzees. Therefore, for each fossil hominin lacking the trapezium (*A. africanus*, *A. sediba*, and the two Swartkrans specimens), we ran the model once with a modern human trapezium, and then with a chimpanzee one, plotting both as different -projected- data points in our PCAs (see legends of Figures 2 and 3). In these cases, the trapezia were scaled so that their articular surface borders corresponded as much as possible to those of the adjoining first metacarpals. As indicated in the last section of Method Details, the overall analytical procedure (including the above trapezium adjustments) was shown to present substantial inter-observer repeatability under blind analytical conditions. Furthermore, our results indicated that the potential error due to trapezium morphology did not influence the observed patterns for each species (i.e., those presented in Figures 2 and 3; also see information in the section below). It should be noted that, even though the trapezium of *A. afarensis* (AL 333-80) likely belongs to a different individual than the one represented by that species’ metacarpal (AL 333-w39), it was used in our biomechanical models after its size was adjusted to correspond to the metacarpal’s adjoining articular surface.

#### Calculating biomechanical efficiency (torque)

Biomechanical efficiency is broadly defined as the degree in which the movement of a musculotendinous unit reflects the theoretical maximum effectiveness.^67^ To quantitatively compare biomechanical efficiency of the opposition of the thumb among different species (Table 1), we use a musculoskeletal model to predict the torque |τ| generated by *m. opponens pollicis* at the TMC joint of the thumb (Figure 1; Tables S2 and S3). Therefore, here we use the terms “biomechanical efficiency” and “torque” as synonyms. We employ a novel modeling approach that integrates muscle parameters and bone 3D morphology, relying on 3D landmarks digitized on the bone surface. These represent muscle origin area, insertion area, as well as the location of the joint (Figure 1B). One of the core novelties of our approach is that we use several landmarks to characterize each enthesis, including three landmark positions at the muscle’s origin enthesis (trapezium tubercle) and three at its insertion area (lateral metacarpal; Figure 1). Our computational muscle model then predicts active forces between each pair of origin and insertion points (i.e., nine possible pairs corresponding to nine torque calculations for each individual / model). The landmark points used in the models are defined in Table S4. It should be noted that, in the *A. afarensis* specimen (A.L. 333-80), the trapezium’s origin enthesis (i.e., the palmar tubercle) is damaged. For this purpose, our models focusing on this specimen relied on a single 3D landmark on the trapezium (i.e., “L4” in Table S4), which was digitized at the most elevated point of the tubercle’s surviving portion. We argue that the resulting torque values are representative of this individual because torque calculations in our sample were found to highly correlate across landmark pairs, despite them involving three distinct locations of the trapezium enthesis (Table S5). This result suggests that interindividual differences in moment arm did not substantially vary by landmark selection on the trapezium’s tubercle (also see descriptive Table S3), encouraging our main statistical analyses to focus on the trapezium landmark point that was also preserved in *A. afarensis* (see below in section “Main statistical analysis”). Moreover, we would argue that the reliability of any attempted (mathematical or geometrical) reconstruction of the tubercle’s missing landmark points would likely be extensively undermined by the very high morphological variability of hand muscle attachment sites (e.g., Karakostis et al.^62^), in combination with the fact that the complete trapezium morphology of this 4-million-year-old species of *Australopithecus* is entirely unknown.^4^

Furthermore, moment arms and torques are influenced by overall size, which varies greatly among hominins (e.g., the hands of *Australopithecus* or *H. naledi* are much smaller than those of *H. sapiens* and Neanderthals). In order to estimate the effects of overall size on our torque calculations, we also ran the models in size-adjusted space, which resulted from uniform scaling of the 3D coordinates (XYZ) of the above-described landmarks (Table S4) to the same centroid size. This technique comprises a standard step for size-adjustment in landmark-based geometric morphometrics.^9^^,^^62^

The first step in constructing each model was to define vectors specifying the location of landmarks in the coordinate frame of the model: Origin o represents the landmark at the trapezium. Insertion i represents the landmark on the first metacarpal (insertion landmark i). The joint position, i.e., the central point of the articular surface, is denoted as j. We assume that the torque generated by *m. opponens pollicis* at the joint can be calculated as the cross product

(Equation 1) $$\vec{\tau }=\vec{\text{r}}\text{×}\vec{\text{F}}\text{M}$$

where r=o-j is the vector of application of the force with respect to the joint. The muscle force vector is the product of a scalar muscle force level FM (in Newtons) predicted by the muscle model and the direction of the muscle force eM with eM=1.

(Equation 2) $${\vec{\text{F}}}_{\text{M}}{=\text{F}}_{\text{M}}{\vec{\text{e}}}_{\text{M}}$$

with the unit vector in the direction of the muscle’s line of action between origin and insertion

$${\vec{\text{e}}}_{\text{M}}=\frac{\vec{i}-\vec{o}}{l_{\text{M}}}$$

and the length of the muscle

(Equation 3) $$l_{\text{M}}=\left|\vec{i}-\vec{o}\right|$$

This approach assumes a straight line of action of the muscle between its origin (trapezium enthesis) and insertion (metacarpal enthesis) sites. Based on the drawn lines of action (Video S1) and bone orientations, for all specimens analyzed in this study, contraction of *m. opponens pollicis* was always associated with flexion at the TMC joint. Importantly, we selected only pairs of entheseal landmarks which could be connected via a straight line without passing through bone (Figure 1B). As *m. opponens pollicis* is the deepest muscle in the area, we assume that there is no other soft tissue possibly blocking this straight line of action. This assumption was also supported by our direct observations during our dissections conducted for a previous human cadaver study focusing on this muscle^41^ as well as by our more recent observations of chimpanzee and human hand skeletons with preserved soft tissue (see in the above section). Nevertheless, it must be highlighted that this is impossible to confirm for fossil hominin specimens, where soft tissue is entirely absent. Therefore, the possibility that the muscle’s line of action in extinct species might have perhaps been shifted by soft tissue constitutes an untestable limitation of our modeling approach.

Hence, all parameters of the joint torque are determined by the locations of the landmarks, except the scalar muscle force FM. The muscle force FM is determined by a Hill-type muscle model 50 (further technical information is provided in the next section below). In this study, the only determining parameter for the muscle force is the maximum isometric force of the muscle at optimal muscle fiber length (Fmax ), which can be calculated from the specific muscle tension σ (a muscle fiber property) and the physiological cross sectional area APCSA (a morphological parameter)

(Equation 4) $${\text{F}}_{\text{max}}\;=\sigma \cdot {\text{A}}_{\text{PCSA}}$$

This approach has been used in a previous anthropological simulation study on *A. afarensis* locomotion^68^ and is a common approach in biomechanics.^69^ For all specimens, we assumed the identical muscle tension of σ=25 Ncm-2,^70^ a value which was also used in previous biomechanical studies on the hand (e.g., previous study^71^).

Even though muscle forces comprise a central component of biomechanical efficiency,^22^^,^^71^ past morphological research on fossil hand bones did not address the potential differences across hominin species in muscle force-generating abilities. However, considering that muscle force-producing capacities are known to vary greatly among great apes and even between humans and chimpanzees,^34^ assessing the manual dexterity of fossil hominins entirely based on bone geometry is prone to severe misinterpretations regarding their manual dexterity.^24^^,^^25^ Our modeling approach addresses this issue by incorporating the factor of muscle physiological cross-sectional areas (PCSA), a proxy of maximum force-generating capacities.^72^ In all analyses, we assumed the mean chimpanzee PCSA^37^ for the chimpanzee group and the mean modern human PCSA^73^ for all modern humans. For Neanderthals, due to their extensive genetic, musculoskeletal, and chronological similarities with modern humans,^18^^,^^38^ a mean modern human PCSA was also assumed, considering that a chimpanzee-like PCSA would be extremely unlikely. Nevertheless, it must be noted that this decision represents a considerable factor of potential bias because soft tissue morphology is unknown in extinct fossil hominins. For the remaining fossil hominins, we ran the models once with a mean human PCSA value (Figures 2A and 3A), and a second time with a mean chimpanzee PCSA value (Figures 2B and 3B). Given the enormous difference between chimpanzees and humans in the mean PCSA of *m. opponens pollicis*, this represents an extreme range of possible PCSA variation. In detail, the four muscle paradigms used were the following:

Paradigm 1 human PCSA: APCSA,human=2.63 cm2 (mean; n = 6) PCSA from a previous study,^73^ who reported a standard deviation of 1.28 cm2, resulting in Fmax =66N.
Paradigm 2: chimpanzee PCSA: APCSA,chimp=1.55 cm2 (mean; n = 4) PCSA from previous research,^37^ who reported a standard deviation of 0.39 cm2, resulting in Fmax =39N.
Paradigm 3: normalized PCSA such that Fmax =1.
Paradigm 4: normalized PCSA scaled by the ratio between human and chimpanzee PCSA such that Fmax =0.59.

As all other parameters of the muscle model are kept constant for the analysis, the four different PCSA paradigms basically result in four different values for the muscle force FM, which then determine the magnitude of the force vector (Equation 2) and influence the joint torque (Equation 1). The values are summarized in Table S2. Please note that there is a small deviation between Fmax and FM due to the internal contraction of the muscle model (see section below).

Hence, there are two sources for the differences in joint torque: a) the geometric difference in origin, insertion and joint landmarks and b) difference in PCSA (human versus chimpanzee).

The above-outlined process for the calculation of biomechanical efficiency (torque) was performed in MATLAB/Simulink (release 2019a), making use of the Simscape Multibody environment for the rigid body calculations (landmark positions, joint positions, and joint torque calculation). The muscle model was implemented in Simulink and is open-source available here (https://github.com/daniel-haeufle/macroscopic-muscle-model). The differential equation for muscle contraction was solved with the ODE15s variable time-step solver, with absolute and relative tolerance set to 1 × 10^−6^. More technical details on the muscle model are provided in the section directly below.

#### Hill-Type muscle model to predict force

As described above, the calculation of biomechanical efficiency (torque) requires the calculation of the muscle force FM. In this study, we used a previously published Hill-type muscle model to calculate FM.^42^

This model consists of four distinct structural elements (see inset of Figure 1B). At the core of the model is the so-called contractile element (CE), which considers the dependency of muscle fiber force FCElCE(t),lCE(t),a(t) on fiber length lCE(t), contraction velocity lCE(t), and muscular activity a(t). The other three elements represent the passive tissue: the elasticity of connective tissue around the muscle fibers (parallel elastic element PEE), the elasticity of the tendon (series elastic element SEE) and the tendon’s viscous damping properties (serial damping element SDE).

With the assumption of force equilibrium between those elements

$${\text{F}}_{\text{CE}}\left(l_{\text{CE}}\text{,}{\dot{l}}_{\text{CE}}\text{,a}\right){+\text{F}}_{\text{PEE}}\left(l_{\text{CE}}\right){=\text{F}}_{\text{SEE}}\left(l_{\text{CE}}\text{,}l_{\text{M}}\right){+\text{F}}_{\text{SDE}}\left(l_{\text{CE}}\text{,}{\dot{l}}_{\text{CE}}\text{,}{\dot{l}}_{\text{M}}\text{,}a\right)$$

it is possible to derive a first order ordinary differential equation describing the internal state of the muscle lCE in dependence of the muscle tendon units length, contraction velocity and the muscular activity: ${\dot{l}}_{\text{CE}}\left(l_{\text{M}}\text{,}{\dot{l}}_{\text{M}}\text{,}l_{\text{CE}}\text{,}a\right)$. By solving this differential equation, the muscle force *F*_*M*_ = *F*_*CE*_ + *F*_*PEE*_ is predicted.

We calculated biomechanical efficiency (torque) in a static hand posture. Therefore, each simulation considers an isometric contraction i.e., at constant muscle length lM, determined by the chosen origin and insertion landmarks (Equation 1; see Table S4). Furthermore, we assumed full muscular activity a = 1 to assess the maximal biomechanical efficiency. The initial condition of the model lCE(t=0) was chosen such that force equilibrium (Equation 2) was fulfilled.^68^

The model requires a set of parameters, most of which are generic.^42^ The main muscle specific parameter which determines the muscle force in this study is the maximum isometric muscle force Fmax, as described in detail in the main text.

The other muscle-specific parameters are the reference lengths of the contractile element lCE,opt and the tendon lSEE,0. These parameters were adapted to the size of the muscle-tendon length to always result in the same ratio γ between muscle contractile element (CE) and tendon (SEE):

$$l_{\text{CE,opt}}=l_{\text{M}\cdot \gamma }$$

$$l_{\text{SEE,0}}=l_{\text{M}}-l_{\text{CE,opt}}$$

The ratio was determined from human cadaveric data with fiber length of lCE,opt=2.29 cm (mean value from previous research,^73^ n = 6, reported standard deviation of 0.62 cm) and the muscle length in our modern human geometry (i.e., a recent modern human individual of our sample) of lM= 4.14 cm, resulting in a ratio of γ=0.55. The torque ${\vec{\tau }}_{\mathrm{m,n}}$ was calculated separately for all possible combinations of origin landmarks m∈(13,18,19) and insertion landmarks n∈(1,2,3). Each combination resulted in different vector of application of the force ${\vec{r}}_{\mathrm{m,n}}={\vec{o}}_{m}-{\vec{j}}_{n}$ rm,n=om-jn and different force vector ${\vec{F}}_{M\;\mathrm{m,n}}$.

This approach has the advantage that, for the static analysis performed here, every landmark pair results in the same muscle force FM for each pair of landmarks.

#### Quantifying 3D bone projection

The developed models focused on the calculation of joint torque based on three landmark locations on the elevated bone area of the metacarpal muscle attachment (Figure 1C; Table S4). In order to further address variability in bone projection across the entire entheseal surface, we analyzed this area using the highly repeatable 3D geometric morphometric approach introduced in previous research^62^^,^^63^ The entire process was carried out using the Geomorph package (version 3.3.1) of the R software.^53^ That previous study focused on a sample from the same recent modern human collection (Basel-Spitalfriedhof collection), identifying a primary principal component associated with proportional elevation across the 3D entheseal surface (also see Karakostis et al.^63^). Here, in addition to the original 45 adult males from the documented Basel-Spitalfriedhof collection,^30^^,^^55^^,^^63^ we included the metacarpal muscle attachments from our fossil sample (Table 1) as well as an additional well-preserved Neanderthal (Chapelle-aux-Saints) and five early modern humans from the Upper Paleolithic (Abri Pataud 1 and 2, Dolni Vestonice 3 and 16, and Arene Candide 2). Detailed information on these fossils’ characteristics is presented in past research on the hand bones.^18^^,^^63^

For defining the bone region of attachment for this muscle, we followed previous research placing the insertion site of *m. opponens pollicis* along the metacarpal’s distalo-lateral ridge.^33^^,^^38^, ^39^, ^40^^,^^63^ It should be noted, however, that some anatomical literature sources report that this insertion site in humans is longer than that, expanding across most of the lateral metacarpal shaft (e.g., Drake et al.^74^). This broader area encompasses both the distalo-lateral roughened area as well as a large amount of surface that does not typically present distinctive alterations on dry bone.^62^^,^^75^ Moreover, this might not consistently be the case for the insertions of chimpanzees (e.g., see Jacofsky et al.^76^), despite the fact that their *m. opponens pollicis* also broadly attaches in the lateral metacarpal shaft.^33^^,^^34^ Nevertheless, based on hand dissections conducted by some of us^41^ (also see Acknowledgments), the extent of this muscle’s attachment site in humans shows extensive variability, sometimes occupying an extremely limited portion of the lateral metacarpal shaft. In fact, the high variability of the extent of muscle attachment sites on human bone surfaces has been frequently reported in the anatomical literature (e.g., see Haładaj et al.^77^ and examples of references therein). On this basis, and considering that soft tissue morphology is unknown in extinct fossil species, the comparative analyses of the present study were restricted to the entheseal structure that was consistently identifiable in dry bone across species (i.e., the distalo-lateral surface roughening in the first metacarpal; Figure 1 and Video S1).

We employed the same landmarking strategy as in our previous geometric morphometric study,^62^ involving six geometrically defined fixed 3D landmarks on the entheseal outline on the bone (see description for landmark points L2, L3, L7, L8, L9, and L10 in Table S4 and Figure 1). These were placed at the attachment’s four most extreme borders (proximal, distal, medial, and lateral) as well as at the two outline angles separating the proximal portion of the enthesis from its distal elongated part (see side images of Figure S1A). The fixed points were used as a basis for calculating a set of 30 equidistant semilandmarks, which were allowed to slide following a minimum Procrustes distance criterion. In agreement with our standard protocols for analyzing entheses,^18^^,^^62^^,^^75^ we made sure that the analyzed entheseal shapes were likely not affected by distinctive pathological or taphonomic effects (i.e., the digitized landmarks were not located on damaged or missing areas). Subsequently, after using Procrustes superimposition to transform the raw 3D landmark coordinates into shape variables (i.e., Procrustes landmark coordinates), we performed a shape principal component analysis (shape PCA). The resulting shape PC1 explained 48.15% of total shape variance and reflected variation in the distribution and degree of bone surface projection across the entheseal area (Figure S1A). Individuals with positive values (recent *Homo* and Swartkrans) showed a relatively higher bone projection than those with negative values (chimpanzees and *Australopithecus*). In *Homo*, the degree of this projection was even relatively higher at the distal portion of the enthesis, near the metacarpal head (see shape changes in Figure S1A).

To further confirm that the degree and distribution of muscle attachment bone projection is in principle associated with biomechanical efficiency (torque) in our models, we performed a series of four multivariate regression analyses (one for each of the four model paradigms). In these analyses, we used proportional bone projection in muscle attachment sites (shape PC1; see Figure 1C and Figure S1A) as a predictor and three of the torque calculations as dependent variables. All of them identified a significant (p < 0.01) and positive correlation between the three torque calculations and a prediction model based on entheseal shape PC1 (explaining 26%–53% of total torque variance in the sample, based on the R2 values; see results in Table S6). These results offer the first biomechanical validation of the traditional concept that the degree of entheseal projection affects biomechanical efficiency (torque)^40^^,^^62^^,^^78^ (see also results in Karakostis et al.^63^). All variables met the necessary statistical assumptions for these tests,^79^ including linearity (based on bivariate plots), no multicollinearity (based on variance inflation factors), residual normal distribution and no outliers (based on z-score distributions), homoscedasticity (based on bivariate plots), and sample size requirements (20 specimens per predictor).

#### Model precision and validation

The precision of our analyses was verified through the application of double-blind analytical procedures involving researchers from three distinct research groups, followed by a double-blind inter-observer repeatability analysis (Figure S1C). Specifically, the 3D surface scans of bones were provided by FAK (University of Tübingen, Germany), virtual positioning of the 3D reconstructions was carried out by IA (Medical School of the National and Kapodistrian University of Athens, Greece), landmark digitization and geometric morphometric analysis was performed by FAK, model development and torque calculations were carried out by DH (Center for Integrative Neuroscience, Germany), and all statistical analyses were conducted by FAK. Prior to this procedure, specimens were assigned a random numeric label before their analysis by DH and IA. For the repeatability analysis, five randomly selected models were used, including *A. afarensis* (composite model), the Neanderthal specimen Shanidar 4 (mirrored bones), the fossil modern human Ohalo 2 (mirrored bones), Swartkrans specimen SK84 (combined with a modern human trapezium), and a recent modern human individual from the Basel-Spitalfriedhof collection. For all these models, FAK performed virtual positioning (instead of IA), whereas IA digitized the landmarks points used in the model and geometric morphometrics (instead of FAK). Subsequently, a new numeric label was assigned to each of the five repetitions and DH ran the models treating them as separate individuals. Finally, FAK calculated the PC scores of these specimens and projected them in the PCAs (Figures 2 and 3), showing that the difference between the repetitions of each model was small and does not affect the patterns observed in this study.

Our study’s resulting difference (%) in mean torque between recent modern humans and chimpanzees closely agrees with that found by previous experimental research for the same joint and muscle.^34^ In the latter study, the average chimpanzee torque was 39.15% of the mean modern human one, while the same proportional difference in our study’s dataset was 43.57%. For that calculation, we computed the average torque of each species by calculating the grand mean of all its nine mean torque calculations (Table S3). This very slight relative difference in the values obtained by the two studies (i.e., by approximately 4.4%) is well within the standard deviations of our mean calculations (Table S3), even though they were based on different samples, methodologies, and formulae for calculating torque.

#### Methodological limitations

Our analysis has limitations which should guide future investigation on this topic. First, the distinctive patterns observed here involve a single muscle, joint, and direction of movement. Even though *m. opponens pollicis* and its contribution to flexion at the TMC joint comprise a vital component of thumb opposition in humans and chimpanzees, other hand muscles, not considered here, also play an important role.^33^ For instance, thumb opposition also involves the coordination of the other thenar muscles: *m. adductor pollicis*, *m. flexor pollicis brevis*, and *m. abductor pollicis brevis*.^27^ In fact, in bonobos, the latter two muscles are reported to be often fused together with *m. opponens pollicis*.^80^ The same two muscles also present relatively large moment arms at the TMC joint^34^ that allows them to be recruited for forceful thumb motion, whereas *m. opponens pollicis* may be considered as more of a dynamic ligament, due to its close proximity to the thumb metacarpal and its oblique orientation.

Our biomechanical models focused on a static hand grip, without incorporating a dynamic approach (e.g., Delp et al.^81^). A future application of the latter would enable an observation of how torque values may vary among different thumb postures throughout the TMC joint’s range of motion, while the range of motion in fossil hominins could be assessed based on ROM predictions (e.g., see study and code provided in Manafzadeh and Gatesy^82^). In such a study design, to account for potential bone interferences within the muscle’s assumed line of action, wrapping surfaces and/or via points could be employed.^83^ Importantly, future research would greatly benefit from defining the exact anatomical position of bones in each model based on anatomical or joint coordinate systems (e.g., see Kambic et al.;^84^ also see Bishop et al.^85^ and associated open-access code at https://doi.org/10.5061/dryad.73n5tb2v9). Despite the verified inter-observer repeatability of the present study’s analytical procedure (Figure S1C), the application of such coordinate systems would likely allow for an easier replicability of the models by other researchers, offering a more semi-automatic definition of joint centers and bone orientation (based on the shape of their adjoining articular surfaces; e.g., see section “Grip selection and model preparation”).

Another limitation stems from the use of mean human or chimpanzee PCSA values as proxies of muscle force.^34^ This compromise was made because soft tissue is not preserved in the fossil record. Nevertheless, given this variable’s high intraspecies variability,^34^ future research would benefit from a systematic study on how different potential PCSA values within each extant species may influence interspecies comparisons of biomechanical efficiency (torque calculations). It must also be emphasized that, since the actual PCSA of each fossil hominin cannot be assessed, it is possible that the PCSA combinations among the species of our early hominin sample were different to those examined here. Given that this is impossible to investigate empirically, we followed the most parsimonious strategy, which was to compare early hominins either under the assumption of a human- or a chimpanzee-like PCSA (i.e., two extremely different mean PCSAs). Evidently, in case that the actual PCSA differences among earlier hominins were extensive, the differences among them in torque would be affected.

Similarly, as also discussed in the main text, one could reasonably hypothesize that muscle PCSA may vary across fossil hominin species by skeletal (body) size. In this study’s early hominin sample, which is mostly composed of unassociated and/or even entirely isolated hand skeletal remains, the only bone element that could be used as a basis for scaling muscle parameters would be the first metacarpal, whose lateropalmar surface also accommodates most of the *m. opponens pollicis*’ length in life.^33^ However, an association between that muscle’s PCSA and first metacarpal size cannot be validated based on the two extant species of our sample (chimpanzees and modern humans), which are known to exhibit remarkably similar mean bone lengths^50^^,^^51^ but excessively different average PCSAs for that muscle.^34^^,^^37^^,^^73^ This is also the case for the samples of this study, as we found no significant difference in first metacarpal length between modern humans and chimpanzees (Mann-Whitney U test’s p value: 0.19). Future research may be able to effectively address this limitation by identifying potential correlations between bone size and the *m. opponens pollicis*’ PCSA as well as by relying on the discovery of more complete fossil hominin postcranial skeletons.

Furthermore, the slight degree of flexion selected for our grip models partly relied on the chimpanzee ranges of motion provided in a previous study.^86^ However, the ranges of motion in that past research may have been affected by the fact that the effects of soft tissue morphology were not taken into account. Therefore, future work employing dynamic modeling approaches would greatly benefit from relying on assessments of range of motion that considered the influence of soft tissue (e.g., van Leeuwen et al.^25^).

Finally, regarding the implications of our results for stone tool use, it should be highlighted that a complete reconstruction of biomechanical efficiency in fossil taxa would also require a consideration of the object’s form as well as position within the hand. The latter would involve calculating the force encountered by joint torque at the point(s) where the thumb presses against the object’s surface. Incorporating the variable effects of tool form and position on hominin grasping efficiency reliably will depend on the development of novel modeling approaches, as well as the discovery of adequately preserved fossil hominin hand skeletons.

### Quantification and Statistical Analysis

To reveal differences in biomechanical efficiency (torque) and associated bone morphology across species, we performed four principal component analyses (PCAs) based on the four above described model paradigms (i.e., human versus chimpanzee muscle PCSA and raw versus size-adjusted models). For all analyses, considering the fact that torque calculations involving the same metacarpal landmark (L1, L2, or L3; see Figure 1A) were highly intercorrelated (all r values over 0.80; Table S5), we used only three of the nine torque variables, so as to reduce the total number of variables used in the PCAs and strengthen the power of the analysis.^79^ These were the three torque calculations based on each of the three first metacarpal landmark points (i.e., landmarks L1, L2, and L3; see Figure 1a; Table S4) and the highest point of the trapezium’s enthesis (i.e., L4; Figure 1A), which was represented in all specimens of the study (on the incomplete trapezium of *A. afarensis*, see information in previous section “Calculation of biomechanical efficiency (torque).” The strong correlation among torques involving the same metacarpal insertion landmark but different trapezium origin landmarks (L4 to L6) suggests that morphological variation in the origin enthesis of the muscle on the trapezium (i.e., the more “steady” element during opposition) is less influential on efficiency than that of its metacarpal insertion enthesis (i.e., the more “moving” element during opposition).

Our PCAs relied on a total of four variables, combining the three above-mentioned joint torque calculations with the scores of shape PC1 from the 3D geometric morphometric analysis of the *m. opponens pollicis*’ metacarpal enthesis (Figure 1; Figure S1A; also see above section of Method Details). Incorporating this variable to our PCAs is crucial because it enables our multivariate approach to consider how bone projection varies across the entire muscle attachment area, in addition to the three specific landmark points sampled for our biomechanical model calculations (see Figures 1C and 2 and 3). Consequently, the distinct interspecies differences identified in our four PCAs arise from a strong shared correlation between the joint torque values calculated in our models (which rely on three points of the elevated enthesis; Figure 1B) and relative bone surface projection over 36 digitized landmark locations of the muscle attachment site (Figure 1C). Prior to the analysis, we verified that the 3D shape variable (shape PC1; Figure S1A) met all basic assumptions for inclusion in the PCAs (see below).

For all four PCAs, a correlation matrix was used due to varying scales among the four variables.^79^ Before performing each analysis, we verified that the datasets presented multivariate normality (based on Doornik and Hansen tests whose p values ranged from 0.20 to 0.77), absence of significant outliers (based on the z-scores approach), and linearity (based on bivariate plots). For our PCAs, a scree-plot approach^79^ recommended a focus only on the first component (PC1), which represented more than 90% of total sample variance (Table S1). Both before and after size-adjustment, all four factor loadings of PC1 (accounting for > 90% of the variance in both cases) were positive and very high for both the torque and the shape PC1 variables (0.86 or above; Table S1), demonstrating the great strength of the observed multivariate pattern despite the relatively small sample size 79. To ensure that the calculation of the components was not affected by the values of species represented by single individuals (and their combinations), our PCAs were calculated from the samples of chimpanzees, modern humans, and Neanderthals. Subsequently, the remaining fossil individuals were projected into the PCA plot (e.g., see Reich et al.,^87^ Mori and Harvati,^88^ Heaton et al.^89^). All statistical analyses were carried out in SPSS (IBM Inc., New York) and PAST.^54^

Additionally, for providing a basic estimate of fingertip force, we calculated the “torque to thumb length ratio” (TTL; see Table S3). This was computed by dividing all torque values by thumb length, which was defined as the summed maximum lengths of the first metacarpal and the two phalanges (in mm). Given that the resulting values presented multiple decimals, for the purpose of clarity, the resulting values were multiplied by 100. In fossil species with unassociated hand bone remains (i.e., *A. afarensis* and *A. africanus*) thumb elements from different individuals were combined.^4^ For *A. afarensis*, the length of the distal phalanx (A.L. 333-159) was taken from the literature.^90^ The only specimens excluded from this procedure were the two Swartkrans first metacarpals (SK84 an SKX5020), which were found in isolation and their genus/species affiliation remains unknown and debated.^4^^,^^12^^,^^13^

## Supplemental information

Document S1. Figure S1 and Tables S1–S6

Document S2. Article plus supplemental information
