# Supplementary material for: Biomechanics of the human thumb and the evolution of dexterity
Source: Curr Biol. 2021 Mar 22;31(6):1317–1325.e8. doi: 10.1016/j.cub.2020.12.041 (PMC7987722; doi:10.1016/j.cub.2020.12.041)
Supplement: Document S1. Figure S1 and Tables S1–S6 [file mmc1.pdf]

Current Biology, Volume *31*

Supplemental Information

Biomechanics of the human thumb and the evolution of dexterity

Fotios Alexandros Karakostis, Daniel Haeufle, Ioanna Anastopoulou, Konstantinos Moraitis, Gerhard Hotz, Vangelis Tourloukis, and Katerina Harvati

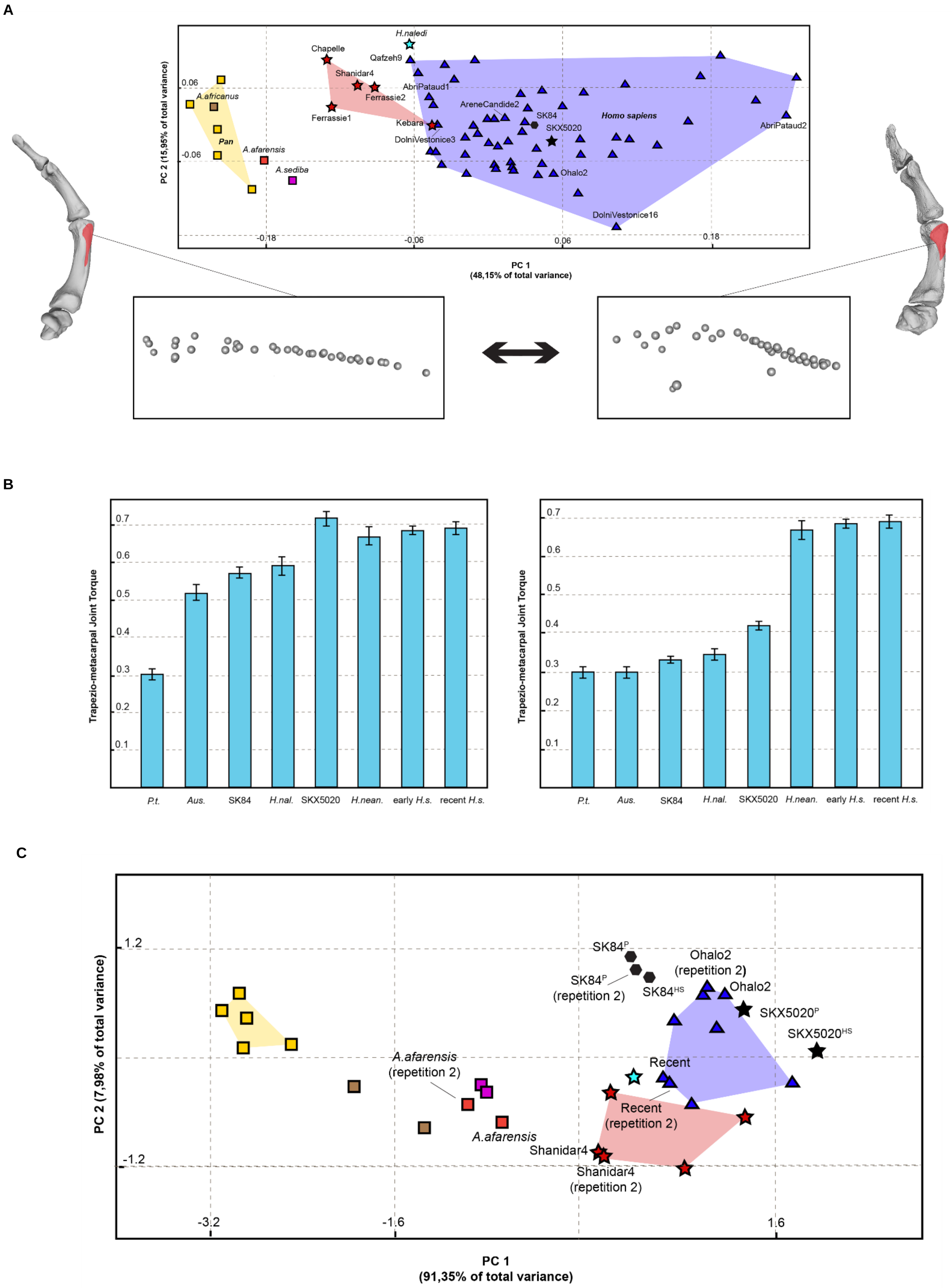
A
PC 2 (15,95% of total variance)
PC 1
(48,15% of total variance)
0.06
-0.06
-0.18
-0.06
0.06
0.18
A.africanus
Pan
A.afarensis
A.sediba
Chapelle
Shanidar4
Ferrassie2
Ferrassie1
H.naledi
Qafzeh9
AbriPataud1
AreneCandide2
SK84
SKX5020
Kebara
DolniVestonice3
Homo sapiens
AbriPataud2
Ohalo2
DolniVestonice16
B
Trapezio-metacarpal Joint Torque
0.7
0.6
0.5
0.4
0.3
0.2
0.1
P.t.
Aus.
SK84
H.nal.
SKX5020
H.nean.
early H.s.
recent H.s.
Trapezio-metacarpal Joint Torque
0.7
0.6
0.5
0.4
0.3
0.2
0.1
P.t.
Aus.
SK84
H.nal.
SKX5020
H.nean.
early H.s.
recent H.s.
C
PC 2 (7,98% of total variance)
1.2
-1.2
-3.2
-1.6
1.6
PC 1
(91,35% of total variance)
SK84P
SK84P
(repetition 2)
SK84HS
Ohalo2
(repetition 2)
Ohalo2
SKX5020P
SKX5020HS
A.afarensis
(repetition 2)
Recent
Recent
(repetition 2)
A.afarensis
Shanidar4
Shanidar4
(repetition 2)

Figure S1. **Composite plot presenting the three-dimensional geometric morphometric analysis of muscle attachment shape (A), graphic summary of torque variables (B), and results of the inter-observer precision test (C). Related to Figures 1 to 3, Table S3, and Video S1.** (A) Plot of the shape principal component analysis on the metacarpal enthesis of *m. opponens pollicis.* The side figures represent 3D shape changes associated with variation along the first principal component, describing proportional bone projection across the muscle attachment site. It should be mentioned that the observed relative projection occurs mostly at the distal portion of the enthesis (closer to the metacarpal head). (B) Bar charts summarizing the sample's variation across groups/specimens in average torque (i.e., "grand mean" values, see Table S3), under the assumption of either a human (left) or a chimpanzee (right) muscle force-generating capacity for the earlier hominins (see Figure 1 and Video S1). Each bar chart indicates the mean value and its standard error, while the respective standard deviations are listed in Table S3. Figure abbreviations: *Australopithecus* (*Aus*.); *Homo naledi* (*H.n.*), Neanderthals (*H.nean*.), *Homo sapiens* (*H.s*.), and *Pan troglodytes* (*P.t*.). (C) Precision analysis: plot of the principal component analysis (muscle paradigm 1) with the projected scores of the five specimens selected for the double-blind inter-observer repeatability analysis. Original and second measurements are labeled in the plot. The difference between the two repetitions do not considerably affect the observed differences among hominin groups. In specimen labels, the superscript "P" indicates that a chimp trapezium was used in the model, while the superscript "HS" refers to the use of a modern human trapezium (see STAR Methods).

Analyses	Eigenvalue	Variance explained (%)	Factor loadings			
**Before size-adjustment**			**13-1**	**13-2**	**13-3**	**sPC1**
**PC1**	3.57	91.35	0.99	0.99	0.98	0.87
**PC2**	0.39	7.98	-0.16	-0.09	-0.19	0.5
**Total**		99.33				
**After size-adjustment**						
**PC1**	3.63	90.66	0.98	0.99	0.98	0.86
**PC2**	0.34	8.55	-0.18	-0.06	-0.21	0.51
**Total**		99.22				

Table S1. Statistics of the four principal component analyses (PCAs), corresponding to the four muscle paradigms utilized (see STAR Methods). Related to Figures 2 and 3.

Paradigm	PCSA	F_{max}	F_M (mean ± standard deviation)
Paradigm 1: Human PCSA	2.63 cm^2	66 N	(63.8±1.4) N
Paradigm 2: Chimpanzee PCSA	1.55 cm^2	39 N	(37.654±0.63) N
Paradigm 3: Normalized human PCSA	-	1	0.966±0.018
Paradigm 4: Normalized chimpanzee PCSA	-	0.59	0.5688±0.0096

Table S2. Summary of the muscle parameters used in the models of the present study. Related to Figure 1.

Group / Specimen	MP	Torques									Grand Mean	TTL
		**L1-L4**	**L2-L4**	**L3-L4**	**L1-L5**	**L2-L5**	**L3-L5**	**L1-L6**	**L2-L6**	**L3-L6**		
Aus.	*H.s*	0.53±0.05	0.50±0.06	0.55±0.05	0.59±0.05	0.56±0.06	0.60±0.05	0.45±0.05	0.42±0.05	0.47±0.05	0.52±0.06	0.67±0.01
	P.t.	0.32±0.03	0.28±0.04	0.34±0.05	0.34±0.03	0.31±0.05	0.35±0.04	0.26±0.03	0.22±0.02	0.29±0.04	0.30±0.04	0.39±<0.01
SK84	*H.s*	0.60±<0.01	0.55±<0.01	0.62±<0.01	0.61±<0.01	0.57±<0.01	0.62±<0.01	0.53±<0.01	0.51±<0.01	0.55±<0.01	0.57±0.04	-
	P.t.	0.35±<0.01	0.32±0.01	0.36±0.01	0.36±0.03	0.33±0.03	0.37±0.03	0.31±0.01	0.29±0.01	0.32±0.01	0.33±0.03	-
SKX5020	*H.s*	0.76±0.07	0.72±0.06	0.75±0.07	0.78±0.01	0.75±0.01	0.76±0.01	0.65±0.07	0.64±0.08	0.64±0.07	0.72±0.06	-
	P.t.	0.45±0.04	0.43±0.03	0.44±0.04	0.46±0.01	0.44±<0.01	0.45±0.01	0.38±0.04	0.37±0.04	0.38±0.04	0.42±0.04	-
H.n.	*H.s*	0.70	0.57	0.69	0.57	0.47	0.56	0.63	0.52	0.61	0.59±0.08	0.76±0.09
	P.t.	0.41	0.34	0.41	0.34	0.28	0.33	0.37	0.30	0.36	0.35±0.04	0.45±0.06
H.nean.	*H.s*	0.74±0.06	0.66±0.07	0.75±0.06	0.73±0.01	0.61±0.05	0.73±0.01	0.63±0.07	0.55±0.08	0.63±0.07	0.67±0.07	0.71+0.07
early *H.s.*	*H.s*	0.70±0.04	0.68±0.02	0.71±0.05	0.72±0.01	0.70±0.01	0.72±0.02	0.65±0.08	0.63±0.06	0.66±0.09	0.69±0.03	0.72+0.03
recent *H.s.*	*H.s*	0.70±0.06	0.69±0.06	0.70±0.06	0.76±0.06	0.73±0.06	0.75±0.06	0.64±0.05	0.62±0.05	0.63±0.05	0.69±0.05	0.74±0.05
P.t.	*P.t.*	0.32±0.03	0.28±0.04	0.34±0.05	0.34±0.03	0.31±0.05	0.35±0.04	0.26±0.03	0.22±0.02	0.29±0.04	0.30±0.04	0.34±0.05

Table S3. Descriptive statistics (means ± standard deviations) for the nine torque variables developed, separated by group and muscle paradigm (MP). Related to Figures 1 and 2. MP involves the muscle force-generating capacity of either *Homo sapiens* ("*H.s.*") or chimpanzees ("*P.t.*"). For facilitating comparisons, the table includes the grand mean (and its standard deviation) for each group / specimen. The latter variable is also graphically summarized in Figure S1B, which includes the grand means' standard error. The rightmost column represents the mean "torque to thumb length index" (TTL), which was calculated by dividing all computed torque values by the corresponding thumb length (in mm) and then multiplying the result by 100 (see STAR Methods). Table abbreviations: *Australopithecus* (*Aus.*); *Homo naledi* (*H.n.*), Neanderthals (*H.nean.*), *Homo sapiens* (*H.s.*), and *Pan troglodytes* (*P.t.*).

Landmark	Bone	Description
**L1**	Metacarpal	Most projecting point of the enthesis
**L2**	Metacarpal	Most proximal point of the enthesis
**L3**	Metacarpal	Most distal point of the enthesis
**L4**	Trapezium	Most projecting central point of the enthesis
**L5**	Trapezium	Midway between the most projecting point and the medial border of the enthesis
**L6**	Trapezium	Midway between the most projecting point and the lateral border of the enthesis
**L7**	Metacarpal	Most palmar point of the enthesis
**L8**	Metacarpal	Most dorsal point of the enthesis
**L9**	Metacarpal	Medial angle separating the enthesis into a proximal elongated portion and a distal tubercle
**L10**	Metacarpal	Lateral angle separating the enthesis into a proximal elongated portion and a distal tubercle

Table S4. Definitions of the fixed 3D landmarks used in the biomechanical models and the geometric morphometric analysis. Related to Figures 1 to 3.

	Torque pairs		r-value	p-value
**Modern human muscle paradigm**	L1-L4	L1-L5	**0.82**	**< 0.001**
	L1-L4	L1-L6	**0.92**	**< 0.001**
	L1-L5	L1-L6	**0.83**	**< 0.001**
	L2-L4	L2-L5	**0.87**	**< 0.001**
	L2-L4	L2-L6	**0.94**	**< 0.001**
	L2-L5	L2-L6	**0.90**	**< 0.001**
	L3-L4	L3-L5	**0.84**	**< 0.001**
	L3-L4	L3-L6	**0.91**	**< 0.001**
	L3-L5	L3-L6	**0.84**	**< 0.001**
**Chimpanzee muscle paradigm**	L1-L4	L1-L5	**0.83**	**< 0.001**
	L1-L4	L1-L6	**0.94**	**< 0.001**
	L1-L5	L1-L6	**0.80**	**< 0.001**
	L2-L4	L2-L5	**0.84**	**< 0.001**
	L2-L4	L2-L6	**0.94**	**< 0.001**
	L2-L5	L2-L6	**0.84**	**< 0.001**
	L3-L4	L3-L5	**0.83**	**< 0.001**
	L3-L4	L3-L6	**0.94**	**< 0.001**
	L3-L5	L3-L6	**0.81**	**< 0.001**

Table S5. **Spearman's bivariate correlations among the nine torque calculations. Related to Figure 1.** Each torque variable is represented by its corresponding pair of landmarks (L) utilized in the models for the muscle's origin and insertion points (see Table S4). The p-values maintained their significance (p-value < 0.05) after Holm-Bonferroni correction.

**Analysis**	**p-value**	**F-value**	**R^2**	**Model / Torque correlations (r-values)**		
				**13-1**	**13-2**	**13-3**
**Modern human muscle paradigm**	<0.01	10.59	0.53	0.74	0.74	0.71
***Pan* muscle paradigm**	0.01	4.79	0.27	0.52	0.54	0.49
**Size-adjusted modern human muscle paradigm**	<0.01	7.17	0.41	0.65	0.66	0.61
**Size-adjusted *Pan* muscle paradigm**	<0.01	7.49	0.26	0.51	0.55	0.46

Table S6. Results of the four multivariate regression analyses (one for each paradigm; see STAR Methods) revealing a strong correlation between three torque calculations and 3D proportional bone projection at muscle attachment surfaces. Related to Figure 1. The presented statistics include statistical significance of the model (p-value and F-value), proportion of torque variance explained by entheseal projection (R^2), and correlation coefficients between the predictive model and each torque variable.
